# Prevention of ventilator-associated pneumonia by noble metal coating of endotracheal tubes: a multi-center, randomized, double-blind study

**DOI:** 10.1186/s13613-021-00961-y

**Published:** 2022-01-04

**Authors:** Pierre Damas, Caroline Legrain, Bernard Lambermont, Nadia Dardenne, Julien Guntz, Grâce Kisoka, Pierre Demaret, Anne-Françoise Rousseau, Laurent Jadot, Sonia Piret, Didier Noirot, Axelle Bertrand, Anne-Françoise Donneau, Benoît Misset

**Affiliations:** 1 Department of Intensive Care, University Hospital of Liège, Domaine Universitaire de Liège, B-4000 Liege, Belgium; 2 Department of Intensive Care, Mont Legia Hospital, Groupe Santé CHC, B-4000 Liege, Belgium; 3 Department of Biostatistics, University Hospital of Liège, Domaine Universitaire de Liège, B-4000 Liege, Belgium

**Keywords:** Ventilator-associated pneumonia, Endotracheal tube, Noble metal coating, Antibiotic consumption, intubation, Infection

## Abstract

**Background:**

Ventilator-associated pneumonia (VAP) causes increased mortality, prolonged hospital stay and increased healthcare costs. Prevention of VAP in intensive care units (ICUs) is currently based on several measures, and application of noble metal coating on medical devices has been shown to inhibit the bacterial adherence of microorganisms to the surface. The objective of this study was to evaluate the potential benefit of noble metal coating of endotracheal tubes for the prevention of VAP.

**Methods:**

This was a multi-center, randomized, controlled, double-blind, prospective study including ventilated patients from nine ICUs from four hospital sites in Belgium. Patients were randomly intubated with identical appearing noble metal alloy (NMA) coated (NMA-coated group) or non-coated (control group) endotracheal tubes (ETT). Primary endpoint was the incidence of VAP. Secondary endpoints were the proportion of antibiotic days during ICU stay and tracheal colonization by pathogenic bacteria.

**Results:**

In total, 323 patients were enrolled, 168 in the NMA-coated group and 155 in the control group. During ventilation, VAP occurred in 11 patients (6.5%) in the NMA-coated group and in 18 patients (11.6%) in the control group (*p*  = 0.11). A higher delay in VAP occurrence was observed in the NMA-coated group compared with the control group by Cox proportional hazards regression analysis (HR 0.41, 95% CI 0.19–0.88, *p*  = 0.02). The number of antibiotic days was 58.8% of the 1,928 ICU days in the NMA-coated group and 65.4% of the 1774 ICU days in the control group (*p*  = 0.06). Regarding tracheal colonization, bacteria occurred in 38 of 126 patients in the NMA-coated group (30.2%) and in 37 of 109 patients in the control group (33.9%) (*p*  = 0.57).

**Conclusions:**

This study provides preliminary evidence to support the benefit of noble metal coating in the prevention of VAP. A confirmatory study in a larger population would be valuable.

*Trial registration*: Clinical trial number: NCT04242706 (http://www.clinicaltrials.gov)

## Background

Ventilator-associated pneumonia (VAP) is the second most common hospital acquired infection among intensive care unit (ICU) patients and usually occurs within 10 days after endotracheal intubation [1–4]. Prevention of VAP in ICUs is currently based on several measures, which vary between geographic regions [5, 6]. These evidence-based prevention strategies include, among others, oral care with antiseptic solutions, continuous removal of bacteria-rich subglottic secretions, or special design and care of the endotracheal tube (ETT) cuff; however, no single strategy eliminates the risk of VAP. Moreover, concerns have recently been raised regarding the possible negative impact on mortality rates of chlorhexidine used as oral antiseptic [7], suggesting that other antimicrobial strategies should be explored [8].

An alternative strategy to reduce the risk of VAP is coating ETTs with antiseptic and antimicrobial compounds such as antibiotics, chlorhexidine, or noble metals [8]. Favorable outcomes on VAP reduction have been demonstrated for instance in three randomized controlled clinical trials of silver-release-based coating of ETTs [9–11]. Using another coating technique, a new ETT coated with a sub-micron layer of noble metal alloy (NMA) of gold, silver and palladium firmly attached to the surface without significant release into the body has been developed (Bactiguard Infection Protection, BIP). The galvanic effect of noble metal alloys creates a microcurrent that reduces the formation of bacterial biofilm, and thus colonization of the ETTs, and subsequent respiratory infection [12]. Metal-coated urinary catheters have been on the market since 1995 and have been tested in a large number of clinical studies; results have demonstrated a reduction in urinary tract infections and antibiotic use [13–18]. Although there is substantial clinical evidence demonstrating that the NMA coating can reduce the infection rate related to other medical devices such as urinary catheters, more data on the efficacy of NMA-coated ETTs on VAP are needed. Therefore, the aim of this study was to assess the VAP incidence when using NMA-coated compared with non-coated subglottic suctioning ETTs in ICU patients.

## Methods

From November 2, 2018, to January 14, 2019 and from June 16, 2019 to March 3, 2020, patients hospitalized in six ICUs from two sites of the University Hospital of Liège in Belgium (CHU) and in three ICUs from two sites of the Centre Hospitalier Chrétien (CHC) of Liège in Belgium, and expected to require mechanical ventilation for more than 24 h, participated in the study. The study was approved by both institutional ethics committees of CHU Liège and CHC Liège Belgium (B707201836287; 2018/136). The study was registered at ClinicalTrial.gov (NCT04242706). Inclusion criteria were patients aged  ≥ 18 years, intubation with an NMA-coated or non-coated ETT, and an anticipated duration of ventilation of more than 24 h. Exclusion criteria were patients participating in another study or having already participated in this study during the same hospitalization. Patients already ventilated for a short period of time with a conventional ETT were not excluded. However, in cases of accidental extubation or reintubation during ventilation with a study tube, only the first part of ventilation was considered for the study. Informed consent was obtained from each patient or their nearest relative.

Patients were randomized into two groups: NMA-coated group (experimental) with noble metal-coated tubes [BIP Endotracheal Tube Evacuation (BIP ETTEvac), Bactiguard, Tullinge, Sweden], and control group without metal-coated tubes (Endotracheal Tube with Evacuation Lumen, Well Lead Medical Co., Ltd., Guangzhou, China). Randomization was performed by mixing the tubes in boxes of ten before distribution in the ICUs and in the emergency rooms where patients could be intubated. No tubes were available in the operating rooms, because patients intubated for a short period of time did not belong to the target group. The tube package was labeled with a code given by the manufacturer which was kept blinded to the clinicians until the end of the study. Coated and non-coated tubes were totally identical and indistinguishable.

### VAP bundle

A VAP bundle has been implemented in each ICU since 2009 in CHU and 2015 in CHC. It involves semi-recumbent position of at least 30°, oral care, teeth brushing with chlorhexidine 0.2% (Corsodyl, Nottingham, United Kingdom), continuous control of the ETT cuff pressure between 20 and 30 cm H_2_O, and daily assessment of sedation. Continuous subglottic suction has also been implemented in the 3 ICUs from CHC since 2015 and was implemented in the 6 other ICUs in CHU for this study. From 2010 to 2017 the VAP rate fluctuated between 12 and 18 VAP/1000 ventilatory days in CHU.

### Diagnosis of VAP

All randomized patients were treated according to normal clinical routine by the physicians in charge in the respective ICUs. Final diagnosis of VAP was made by one intensivist expert in infectious disease (PD), blinded to the study and not involved in daily clinical work. The clinical suspicions of VAP, leading to antibiotic prescription, were assessed by the usual criteria from the clinical pulmonary infection score [19], in conjunction with those from the ventilator-associated conditions [20], which allowed VAP to be defined as follows: new infiltrate on chest radiography associated with at least fever (> 38.3 °C) or hypothermia (< 36 °C), leukocytosis (> 11.10^9^ white blood cells (WBC)/L) or leukopenia (< 4.10^9^ WBC/L), and at least one of the two last criteria, purulent tracheal secretions confirmed by microscopic examination or worsening of oxygenation defined by the elevation of FiO_2_ of at least 0.2 or of positive end-expiratory pressure (PEEP) level of at least 3 cm H_2_O for 2 consecutive days. In addition, VAP had to be confirmed by a quantitative bacterial culture of 10^6^ CFU/mL of a true pathogen from an endotracheal specimen or of 10^4^ CFU/mL from broncho-alveolar lavage fluid. Only pneumonias occurring after 48 h of ventilation with a study ETT were considered as VAP.

### Data collection

The following data were recorded: age, sex, Charlson score, primary reason for ICU admission, Simple Acute Physiologic Score (SAPS) II and Sequential Organ Failure Assessment (SOFA) score on the day of intubation, presence of infection on the day of intubation, daily antibiotic use from intubation until ICU discharge or until 28 days after intubation, ventilation before or after intubation, reintubation rate, tracheostomy rate, other diagnosis of infections, length of ICU and hospital stay, ICU and hospital mortality. Tracheal colonization was also recorded within 48 h after intubation and then twice a week during ventilation by quantitative culture.

### Endpoints

The primary endpoint was the occurrence of VAP during ventilation in the experimental group and in the control group. Time-to-VAP was also considered. Secondary endpoints were the proportion of antibiotic days during ICU stay and tracheal colonization by pathogenic bacteria.

### Statistical analysis

Quantitative data were summarized as median and interquartile range (Q1–Q3) or as mean and standard deviation (SD) when normally distributed. Experimental and control groups were compared by Mann–Whitney *U* or Student’s *t* test as appropriate for continuous variables and by Chi-square or Fisher’s exact test for categorical variables. VAP-free survival curves were displayed graphically by Kaplan–Meier method and analyzed by Cox proportional hazards regression. Hazard ratios (HR) with 95% confidence intervals (CI) were also calculated. The Fine–Gray competing risk regression model was also computed to consider the competing risk of death. Negative binomial regression with an offset for the number of ICU days were used to compare the number of days of antibiotic therapy in the two randomized groups. All tests were two-sided and statistical significance was set as *p*  < 0.05. The statistical analyses were carried out using SAS (version 9.4 for Windows) statistical package and RStudio.

This was a pilot study, and 300 patients were considered to be sufficient to evaluate the possible effect of the study tubes before a confirmatory study with a higher power. This number was based on prior studies demonstrating the effect of subglottic suction.

## Results

The study started in November 2018 and was interrupted on January 14, 2019, due to a serious adverse event. This event was a tracheal rupture after intubating a patient suffering from acute respiratory failure, pulmonary cancer and acute bacterial pneumonia. The ethics committee asked to stop the study and requested an investigation of the case. After acknowledging that the tracheal rupture was not caused by a defect in the ETT study device nor its quality or performance, the ethics committee allowed the study to restart in May 2019.

### Patient characteristics

**Fig. 1:**
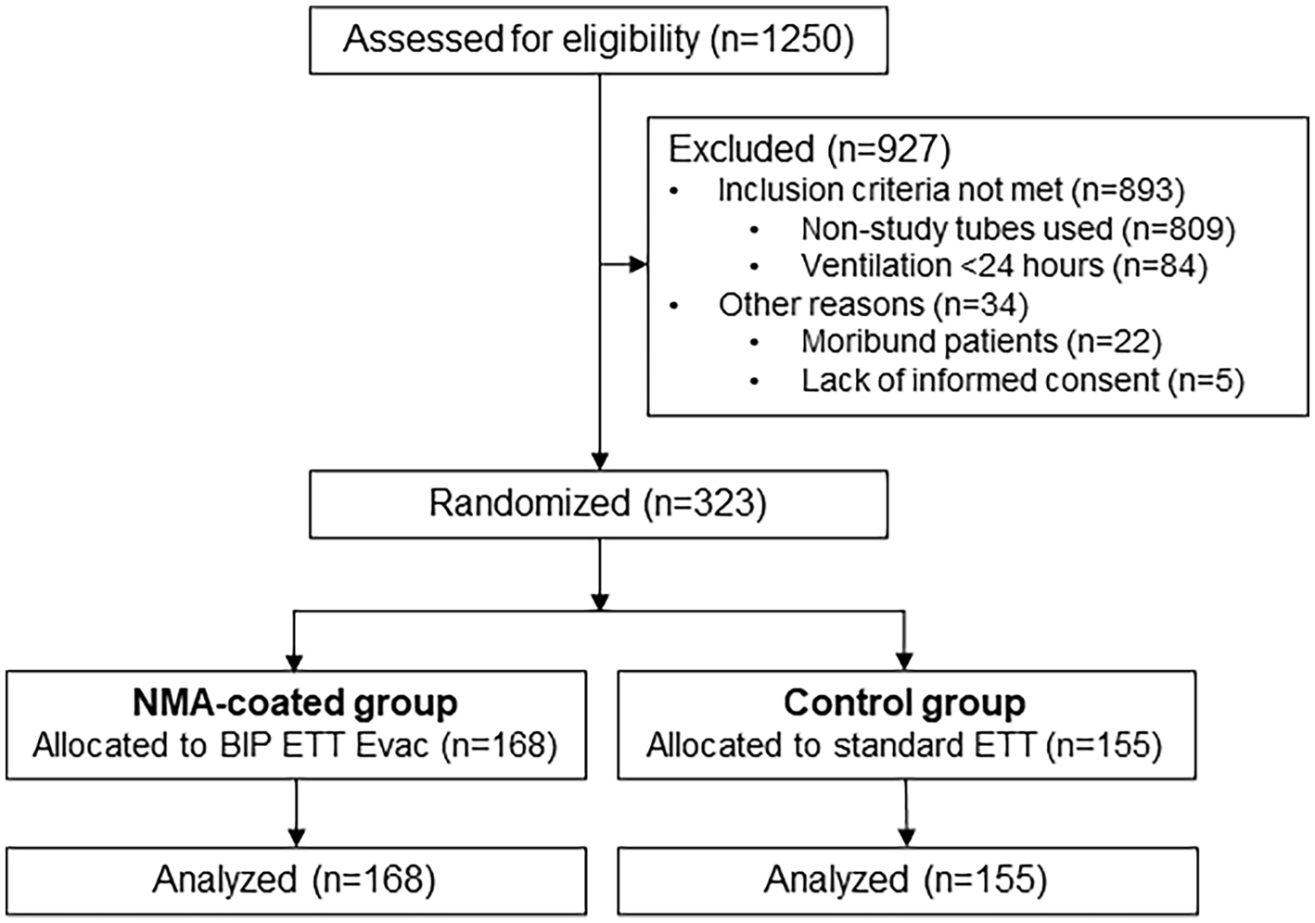
Flowchart of patients admitted in the participating intensive care units during the study. *BIP* Bactiguard Infection Protection; *ETT* endotracheal tube; *Evac* evacuation; *NMA* noble metal alloy

During the study period, 323 patients were randomized, 168 to the NMA-coated group (52%) and 155 to the control group (48%) (Fig. 1). Patient characteristics are presented in Table 1. No differences were observed with respect to age, sex, comorbidities as assessed by Charlson score, type of patients, cause of admission, SAPS II score and SOFA score at admission. One hundred nineteen patients (70.8%) in the NMA-coated group and 121 patients (78.1%) in the control group (78.1%) received antibiotics on the day of the study tube insertion (*p*  = 0.14). Respiratory infections were more frequent (47% vs 38.7%) and intra-abdominal infections were less frequent group (3% vs 11%) in the NMA-coated group (*p*  = 0.048). The categories of antibiotics administered were similar in the two groups. The characteristics of the patients in terms of ventilatory support were similar in the two groups (Table 2). Specifically, 19 patients were ventilated through conventional tubes before intubation with a study tube in the NMA-coated group and 23 patients in the control group. The median duration of mechanical ventilation with the study tube was 5 days in both groups.

**Table 1:** Baseline patient characteristics

Patient characteristics	NMA-coated group	Control group	*p* value
Number	168	155	
Gender female, *n* (%)	75 (44.6)	69 (44.5)	0.98
Age in years, median (Q1–Q3)	67.9 (56.4–74.7)	67.3 (58–74.5)	0.98
Type of patients, *n* (%)			0.58
Medical	139 (82.7)	127 (82)	
Surgical	24 (14.3)	20 (12.9)	
Trauma	5 (3)	8 (5.2)	
Reason for admission, *n* (%)			0.13
Neuro	39 (24.7)	21 (14.7)	
Cardiac	21 (13.3)	29 (20.3)	
Respiratory	53 (33.5)	46 (32.2)	
Digestive	18 (11.4)	25 (17.5)	
Kidney	2 (1.3)	5 (3.5)	
Sepsis	15 (9.5)	10 (7)	
Other	10 (6.3)	7 (4.9)	
Charlson score, median (Q1–Q3)	2 (1–4)	2 (1–4)	0.77
SAPS II, median (Q1–Q3)	48 (38–60)	48 (40–62)	0.85
SOFA 1st day, median (Q1–Q3)	8 (6–10)	8 (6–11)	0.06
Antibiotic therapy on intubation, *n* (%)	119 (70.8)	121 (78.1)	0.14
Reason, *n* (%)			0.048
Central nervous system infection	3 (1.8)	3 (1.9)	
Respiratory infection	79 (47)	60 (38.7)	
Intra-abdominal infection	5 (3)	17 (11)	
Urinary tract infection	6 (3.6)	7 (4.5)	
Sepsis	14 (8.3)	13 (8.4)	
Soft tissue infection	3 (1.8)	2 (1.3)	
Prophylaxis	4 (2.4)	6 (3.9)	
Other	5 (3)	13 (8.4)	
Type of antibiotic therapy given on intubation, *n* (%)			0.10
None	53 (31.6)	35 (22.6)	
Amoxycillin–clavulanic acid	25 (21)	27 (22.3)	
Third generation cephalosporin	16 (13.5)	29 (24)	
Large spectrum antibiotics^a^	62 (52.1)	54 (44.6)	
Other^b^	12 (10.1)	10 (8.3)	

^a^Carbapenem, piperacillin–tazobactam, cefepime

^b^Flucloxacillin, vancomycin, fluoroquinolone, trimethoprim–sulfamethoxazole, amoxycillin

*Q* quartile; *SAPS* Simplified Acute Physiology Score; *SOFA* Sequential Organ Failure Assessment

**Table 2:** Intensive care unit stay characteristics

ICU stay characteristics	NMA-coated group	Control group	*p* value
Hospital days before ICU admission	0 (0–3)	1 (0–6)	0.06
ICU days before intubation with study tubes	0 (0–2)	0 (0–3)	0.12
Ventilated patients before study tube insertion, *n* (%)	19 (11.3)	23 (14.8)	0.35
Ventilation days with conventional tubes before intubation with study tubes	0 (0–0)	0(0–0)	0.35
Ventilation days with study tubes	5 (3–9)	5 (3–8)	0.45
Reintubated patients, *n* (%)	24 (14.3)	26 (16.8)	0.54
Total ventilatory days	5.5 (3–11)	6 (3–10)	0.72
Length of ICU stay, days	11 (6–17)	11 (5–18)	0.79
Antibiotic days during study tube ventilation, *n* (%)	714 (66.7)	657 (73.5)	0.11
Antibiotic days during ICU stay, *n* (%)	1045 (57.9)	1072 (66.7)	0.06
Patients with other infections during ICU stay, *n* (%)	18 (10.7)	19 (12.3)	0.66
ICU mortality, *n* (%)	69 (41.1)	65 (41.9)	0.87
Hospital mortality, *n* (%)	84 (50.0)	80 (51.6)	0.77

All data are presented as median (interquartile range) unless stated otherwise

*ICU* intensive care unit; *NMA* noble metal alloy

### Primary endpoint

During ventilation with the study tubes, VAP was clinically suspected in 22 (13.1%) and 32 (20.6%) patients (*p*  = 0.07) and confirmed in 11 (6.5%) and 18 (11.6%) patients (*p*  = 0.11) in the NMA-coated and control groups, respectively. The incidence density of VAP was 10.5 and 22.4 VAP/1,000 VD in the NMA-coated and control group, respectively (*p*  = 0.07). VAP with impaired oxygenation occurred in 5 patients (3.0%) in the NMA-coated group and in 11 (7.1%) in the control group (*p*  = 0.06).

**Fig. 2:**
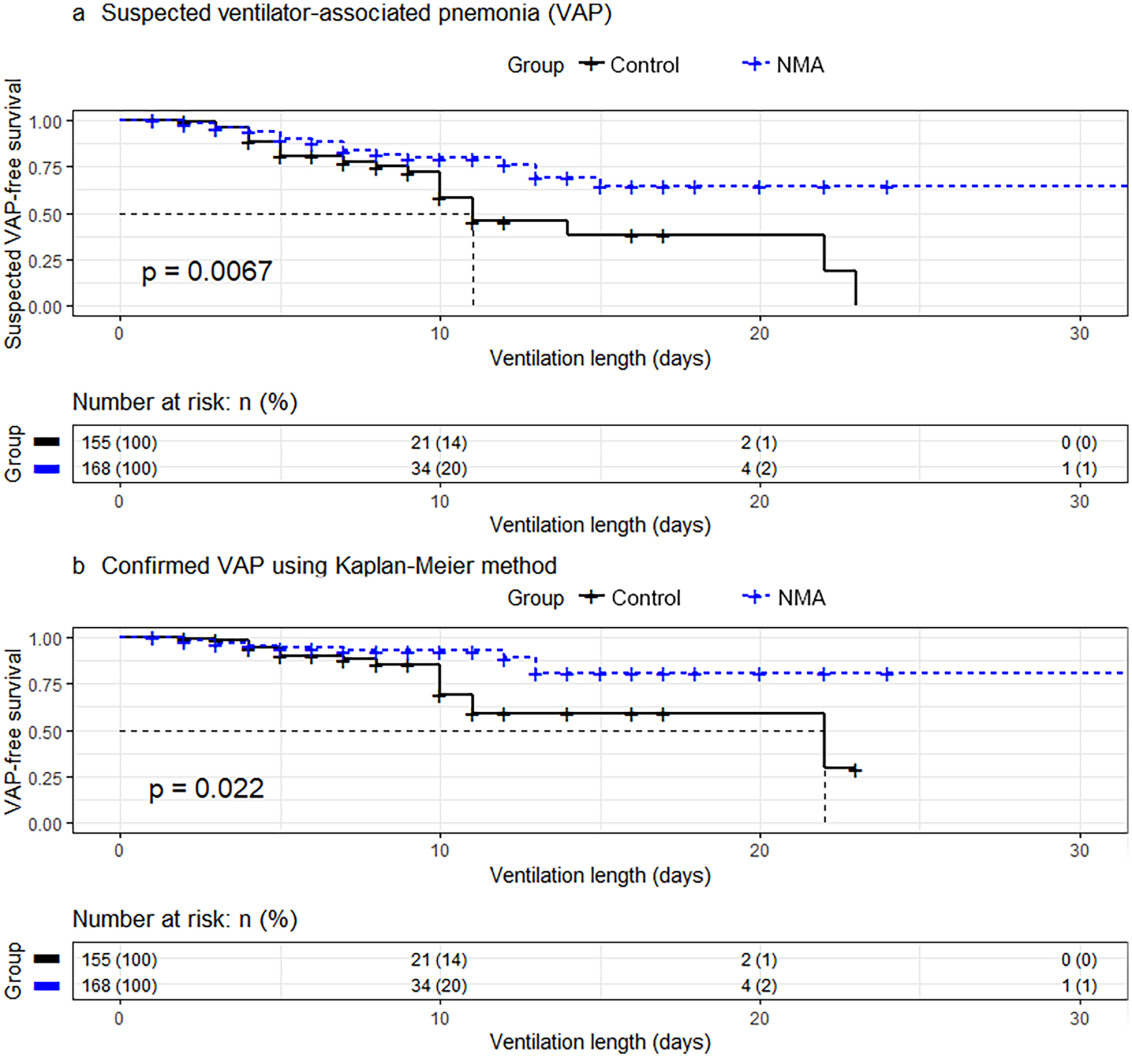
Cumulative rates of patients remaining free of **a** suspected ventilator-associated pneumonia and **b** confirmed ventilator-associated pneumonia using the Kaplan–Meier method

VAP occurred in 12/83 patients (14.5%) who did not receive antibiotics on admission and only in 17/240 patients (7.1%) receiving antibiotics (*p*  = 0.07). As the two treatment groups were homogeneous, no other potentially confounding factors were added in the Cox regression model. Using Cox proportional hazards regression analysis, when considering the antibiotic administration on admission, the time to VAP suspicion and confirmed VAP was higher in the NMA-coated group (HR 0.47, 95% CI 0.27–0.81, *p*  = 0.007 and HR 0.41, 95% CI 0.19–0.88, *p*  = 0.02, respectively) (Fig. 2; Table 3). The Fine–Gray model with death as a competing event showed similar results (Table 3). Similar results were observed in the subgroup of patients who had not been ventilated with a conventional tube prior to inclusion (*n* =  281, HR 0.42, 95% CI 0.18–0.97, *p * = 0.04). Considering the relative uncertainty of VAP diagnosis, the same analysis was done with a diagnosis of VAP including impaired oxygenation. The results were similar and are shown in Table 4.

**Table 3:** Cox and Fine–Gray regression models to evaluate risk of type of endotracheal tubes on ventilator-associated pneumonia suspicion and confirmation

Variable		Cox model		Fine–Gray model	
		HR (95% CI)	*p* value	HR (95% CI)	*p* value
VAP suspicion					
Type of ETTs	Coated	0.47 (0.27–0.81)	0.007	0.53 (0.31–0.90)	0.02
Antibiotic on intubation	No	3.16 (1.83–5.48)	< 0.0001	3.11 (1.81–5.32)	< 0.0001
Occurrence of VAP					
Type of ETTs	Coated	0.41 (0.19–0.88)	0.02	0.47 (0.23–0.99)	0.049
Antibiotic on intubation	No	3.49 (1.66–7.36)	0.001	3.23 (1.60–6.55)	0.001

*CI* confidence interval; *ETT* endotracheal tube; *HR* hazard ratio; *VAP* ventilator-associated pneumonia

**Table 4:** Cox and Fine–Gray regression models to evaluate risk of type of endotracheal tubes on ventilator-associated pneumonia with impaired oxygenation

Variable		Cox model		Fine–Gray model	
		HR (95% CI)	*p* value	HR (95% CI)	*p* value
VAP suspicion					
Type of ETTs	Coated	0.25 (0.09–0.73)	0.01	0.32 (0.12–0.88)	0.03
Antibiotic on intubation	No	1.77 (0.62–5.03)	0.29	1.65 (0.59–4.58)	0.34
Occurrence of VAP					
Type of ETTs	Coated	0.27 (0.09–0.80)	0.02	0.35 (0.13–0.97)	0.04
Antibiotic on intubation	No	1.94 (0.67–5.60)	0.22	1.80 (0.64–5.05)	0.27

*CI* confidence interval; *ETT* endotracheal tube; *HR* hazard ratio; *VAP* ventilator-associated pneumonia

VAP occurrence increased with ventilation duration and ICU length of stay, but was not related to mortality. The median duration of ventilation duration was 12 (6–22) days in patients developing VAP and 4 (2–8) days in patients not developing VAP (*p*  < 0.001). The median length of stay in the ICU of these two groups was 23 (13–33) and 10 (6–17) days, respectively (*p*  < 0.001). The hospital mortality was 48.3% and 51% for VAP and non-VAP patients, respectively (*p*  = 0.55).

### Secondary endpoints

#### Antibiotic consumption

From intubation with the study ETT until ICU discharge or until 28 days after intubation, antibiotic therapy was given for a median of 6 (2–9) days in the NMA-coated group and 6 (3–11) days in the control group (*p*  = 0.43). A treatment with antibiotics was started after intubation in 68 (40.5%) patients of the NMA-coated group and 69 (44.5%) patients of the control group (*p*  = 0.06). A new antibiotic therapy was given for a median of 0 (0–4) days in the NMA-coated group and 0 (0–6) days in the control group (*p*  = 0.28).

#### Bacterial colonization

Tracheal sampling was obtained in 126 patients (75%) in the NMA-coated group and in 109 patients (70.3%) in the control group after 48 h of ventilation. Only qualitative cultures could be obtained, and these were positive in 62 patients (49.2%) in the NMA-coated group and in 51 patients (46.8%) in the control group (*p*  = 0.60). Among these bacteria, 24 were already present in the NMA-coated group and 14 in the control group in tracheal aspirates taken during the first 48 h of ventilation. Thus, new occurrence of bacteria occurred in 38 patients in the NMA-coated group (30.2%) and in 37 patients in the control group (33.9%) (*p*  = 0.57). The delay between intubation and occurrence of bacteria in tracheal aspirates was 4 (3–7) days in the NMA-coated group and 4 (3–6) days in the control group (*p*  = 0.77). Table 5 displays the microorganisms associated with VAP in both groups.

**Table 5:** Microorganisms associated with the diagnosis of ventilator-associated pneumonia in NMA-coated and control groups according to the presence of antibiotic treatment the first day of intubation

Antibiotic treatment	NMA-coated group (*n*)	Control group (*n*)
Yes	*Hafnia alvei *(1)	*Streptococcus pneumoniae* (1)
	*Escherichia coli* (1)	*Enterobacter cloacae* (2)
	*Pseudomonas aeruginosa* (2)	*Enterobacter aerogenes* (2)
		*Citrobacter freundii* (1)
		*Proteus vulgaris* (1)
		*Morganella morgani* (1)
		*Klebsiella pneumoniae* (2)
		*Acinetobacter* (1)
		*Pseudomons aeruginosa* (2)
		*Stenotrophomonas* (1)
No	*Haemophilus influenzae* (1)	*Staphylococcus aureus* (1)
	*Staphylococcus aureus* (3)	*Enterobacter cloacae* (1)
	*Escherichia coli* (1)	*Serratia marcescens* (1)
	*Klebsiella pneumoniae* (1)	*Pseudomonas aeruginosa* (2)
	*Morganella morganii* (1)	

*NMA* noble metal alloy

### Outcome

The length of the ICU-stay, duration of mechanical ventilation, and tracheostomy rate were not significantly different between the two groups. ICU mortality was 41.1% and 41.9% in the NMA-coated and control groups, respectively (*p*  = 0.87), and hospital mortality was 50.0% and 51.6%, respectively (*p*  = 0.77).

## Discussion

In this study, we assessed the efficacy of NMA-coated ETT through a randomized blinded trial. We found that the delay in VAP occurrence was significantly higher in the NMA-coated group of patients compared with the control group, and we also observed a downward trend in antibiotic consumption in this group.

Although a previous study has been conducted with NMA-coated BIP ETT [21], this is the first study testing the NMA coating of ETTs with subglottic suctioning. The double-blind design, which is rare in such studies on VAP prevention, and the multi-center characteristics of the study add robustness to support the effectiveness of NMA coating for the prevention of VAP. One difficulty in performing studies on VAP is determining the diagnosis and it is recommended to consider more objective outcomes when evaluating the potential merits of a specific strategy [22]. Since we used one blinded reviewer to determine the diagnosis of VAP, we believe we were able to control some of this uncertainty.

Antibiotic administration for any reason on the day of intubation also appeared to be particularly efficacious to reduce the occurrence of VAP. However, the effect of metal coating of ETTs still remained significant. Moreover, the control group included a higher proportion of patients receiving antibiotics on the day of intubation and, despite this, had the highest VAP rate.

The VAP rate of the control group (22.4/1,000 VD) may appear high compared with other studies on VAP prevention, which have reported half this number for patients ventilated through ETTs allowing subglottic secretion suctioning [23, 24]. However, it should be emphasized that in those studies the duration of ventilation was higher, the median being 7 days. In terms of ventilated patients, the rate of those developing VAP in the control group was 11.6% in our study, a very plausible number. It should be noted that a reduction in ventilation duration is the effect of an implemented VAP prevention bundle, which was followed in both institutions [25]. Even if VAP was associated with prolonged mechanical ventilation and ICU stay, there was no difference between groups in either endpoint. Obviously, there was a lack of power here because VAP was encountered only in a minority of patients. However, there was also no difference in mortality between VAP and non-VAP patients, a fact already observed in other studies, likely because the attributable mortality of VAP is now considered as very low [22]. For instance, no differences were observed in duration of ventilation, ICU length of stay or mortality in both studies demonstrating the effect of subglottic suction on the incidence of VAP [23, 24].

A recent study reported that patients critically ill patients with COVID-19 are at high risk of developing hospital-acquired infections (HAIs), with 46% of COVID-19 patients admitted to ICU developing HAIs. VAP was one of the most frequent HAIs occurring in 50% of these patients [26]. Therefore, additional strategies for the prevention of VAP are critical to prevent prolonged mechanical ventilation and ICU stay, and increased mortality typically observed in infected patients.

An important aspect to consider is the mechanism of action of the NMA coating in preventing VAP. To note, the bacterial colonization of the ETTs, and subsequently of the trachea, were not different between the NMA-coated and control groups in terms of percentage of patients or delay of these patients to develop a new bacterial colonization in the tracheal sputum. Regarding this, it should be acknowledged that bacterial cultures were only qualitative, which is an obvious weakness of the study. Quantitative cultures could have led to a different result, but this is still to be performed. Additionally, a metabolic effect on bacteria of the microcurrent generated by the metal coating could be hypothesized. This metabolic effect could alter the virulence and biofilm producing ability of the bacteria and may explain the reduction in the VAP rate. One previous study on the NMA-coated ETTs demonstrated a reduction of high-grade biofilm on the surface, which at least could indicate a shift in some aspects of biofilm formation [12]. Further studies are needed to elucidate the microbial mechanisms of the coating.

The main limitation of our study was the limited study population size, which would lead to a lack of power to detect potential differences in secondary endpoints between groups. Another limitation is that bacterial cultures were only qualitative. Differences in bacterial colonization of the ETTs between groups may be observed with quantitative cultures. Since this was a pilot study, we believe a larger study will be able to address these issues.

In summary, the blinded design of the study, the Cox proportional hazards regression analysis, the effect of antibiotic therapy the day of intubation not preventing the excess of VAP in the control group are valuable arguments for the effect of NMA coating in prevention of VAP. This was still true considering the VAP with impaired oxygenation only. However, no other objective data, such antibiotic consumption or colonization rate could confirm this finding.

## Conclusion

This double-blind, randomized study provides preliminary evidence to support the benefit of NMA coating of ETTs in reducing the incidence of VAP in ventilated ICU patients. Given the existing problem of bacterial resistance worldwide, a physical approach to reduce the bacterial colonization of valuable foreign bodies, such as ETTs, is worth further investigation. A confirmatory study in a larger population would be valuable.

## Data Availability

The datasets analyzed during the current study are available from the corresponding author on reasonable request.

## Abbreviations

BIP: Bactiguard infection protection
CHC: Centre Hospitalier Chrétien
CHU: University Hospital of Liège
CI: Confidence interval
ETT: Endotracheal tube
HAI: Hospital-acquired infection
HR: Hazards ratio
ICU: Intensive care unit
IQR: Interquartile range
NMA: Noble metal alloy
PEEP: Positive end-expiratory pressure
SAPS: Simple Acute Physiologic Score
SD: Standard deviation
SOFA: Sequential Organ Failure Assessment
VAP: Ventilator-associated pneumonia
VD: Ventilator-day
WBC: White blood cell
